# Quantifying the Detrimental Impacts of Land-Use and Management Change on European Forest Bird Populations

**DOI:** 10.1371/journal.pone.0064552

**Published:** 2013-05-21

**Authors:** Amy S. I. Wade, Boris Barov, Ian J. Burfield, Richard D. Gregory, Ken Norris, Simon J. Butler

**Affiliations:** 1 Centre for Agri-Environmental Research, School of Agriculture, Policy and Development, University of Reading, Reading, United Kingdom; 2 BirdLife Europe, Brussels, Belgium; 3 BirdLife International, Cambridge, United Kingdom; 4 The Royal Society for Protection of Birds, The Lodge, Sandy, Bedfordshire, United Kingdom; 5 School of Biological Sciences, University of East Anglia, Norwich, United Kingdom; niversity of Marburg, Germany

## Abstract

The ecological impacts of changing forest management practices in Europe are poorly understood despite European forests being highly managed. Furthermore, the effects of potential drivers of forest biodiversity decline are rarely considered in concert, thus limiting effective conservation or sustainable forest management. We present a trait-based framework that we use to assess the detrimental impact of multiple land-use and management changes in forests on bird populations across Europe. Major changes to forest habitats occurring in recent decades, and their impact on resource availability for birds were identified. Risk associated with these changes for 52 species of forest birds, defined as the proportion of each species' key resources detrimentally affected through changes in abundance and/or availability, was quantified and compared to their pan-European population growth rates between 1980 and 2009. Relationships between risk and population growth were found to be significantly negative, indicating that resource loss in European forests is an important driver of decline for both resident and migrant birds. Our results demonstrate that coarse quantification of resource use and ecological change can be valuable in understanding causes of biodiversity decline, and thus in informing conservation strategy and policy. Such an approach has good potential to be extended for predictive use in assessing the impact of possible future changes to forest management and to develop more precise indicators of forest health.

## Introduction

The majority of European forests are heavily influenced by human management, principally for timber production, with just 4% categorised as undisturbed [1]. As a consequence of increasing intensification to improve yields, many forests are becoming more fragmented, much younger and far more homogenous than they would naturally be [2]. It has been repeatedly demonstrated that forests managed for timber production generally have lower biodiversity than undisturbed forest [2], [3], [4], [5], [6], [7], [8], [9], with forest specialists being particularly vulnerable [10], [11], [12], [13], but these changes are likely to further threaten the wealth of biodiversity European forests still support [8].

At its most intensive, forest management entails scarifying the soil, the planting of a single species, extensive thinning, chemical application, suppression of natural disturbance events, and eventual clear cutting. Whilst there is considerable variation in forest management across Europe [14], most involves aspects of this process and, as a consequence, high production, even-aged monocultures are widespread [15]. Our understanding of the ecological effects of the continued intensification of this process in managed forests, both in Europe and elsewhere, is limited compared to the effects of intensification within other production systems, particularly agroecosystems, as the nature and scale of changes are more complex and difficult to quantify. Furthermore, numerous other factors besides intensification, such as declining traditional management [16] and increased deer abundance [17], are also causing stark ecological change in forest systems. Thus, unlike for agriculture [18], a coherent depiction of the impacts of forest change on biodiversity within Europe is lacking. To effectively conserve the diversity of flora and fauna supported by forest systems, across all forest types and successional stages, an improved understanding of the multiple drivers of decline is urgently required.

Birds provide a useful proxy in assessing general biodiversity trends within forest habitats as they are well monitored, sensitive to ecological degradation and have the additional political advantage of public recognition, interest and empathy. Birds have been particularly well monitored in European forests, revealing a consistent decline in populations over recent decades [19]. In this study we present a trait-based framework to assess the risk to European forest birds from changes that have occurred in forest habitats over the same time period. A similar approach has been successfully applied in farmland habitats for birds [20] and other taxa [21] in the UK, and birds at a pan-European scale [22]. Here we extend this approach to more complex forest ecosystems, assessing risk to a wide range of species at a pan-European scale. In doing so we take a pan-European perspective of ecological changes to forest habitats and incorporate multiple drivers of change, which has not previously been achieved. We predicted that those species with a higher proportion of their resources detrimentally affected by changes to forest habitats in recent decades, thus experiencing higher risk, would be those with the most negative population growth rates.

## Materials and Methods

### Pan-European Common Bird Monitoring Scheme

The Pan-European Common Bird Monitoring Scheme (PECBMS: http://www.ebcc.info/pecbm.html) collates population data for 145 European bird species from 25 European countries and generates national, regional and pan-European indices of population growth. The year from which data are available varies between countries and species, ranging from 1966 for many species in the United Kingdom to 2007 for species in Greece and Slovenia. The PECBMS assigns countries into regions based on broad geophysical similarities; here we used data from 20 countries across four regions: North (Finland, Norway and Sweden), West (Austria, Belgium, Denmark, Germany, Netherlands, Ireland, Switzerland and United Kingdom), Central and East (Czech Republic, Estonia, Latvia, Hungary and Poland) and South (France, Italy, Portugal and Spain). Monitoring schemes in the other five countries have not been running long enough to provide sufficient data for the analyses presented here. Regional species indices are calculated as the weighted average of a species' population trend in the constituent countries, weighted by relative breeding population size of each bird species in each country (taken from [23]). Pan-European indices are calculated as the weighted average of regional trends, again based on the relative proportion of the European breeding population found in each region. We used data from 52 species included in the PECBMS in our analyses (Table S1). These comprise species for which >10% of their breeding population use forest habitat according to Tucker and Evans [24], are present in >5 European countries as either breeding or wintering populations [25], and are included in the PECBMS data set from 1980. Data used were pan-European population growth rates of species between 1980 and 2009.

### Quantifying risk in current forest landscapes

The risk assessment framework quantifies risks associated with changes to land-use and compares species' risk to their population growth rate over the same time period. The underlying structure of this framework and the methods employed to quantify risk have been published in detail (see [24] and Appendix S1), as has the method for applying this approach at a pan-European scale [22]. In brief, the risk of forest change *x* to species *y* is defined as the degree of coincidence between the detrimental environmental impacts of that change and the resource requirements of that species, adjusted for the species' ecological resilience, defined by the breadth of its resource requirements and its reliance on forest for those resources. Using these definitions, we developed a risk assessment framework for European forest bird species. Firstly, we constructed a resource requirements matrix for the 52 species by gathering data on their summer and winter diets, summer and winter foraging habitat and nest site location (Table S2, [25]). As in Butler *et al*. [22], species' reliance on forest habitats to provide their key resources was scored by a number of ornithological experts in each country from which PECBMS data were used. Species were scored as having either a major, moderate or minor reliance on forest habitat, or as not being present as a breeding species. The modal response for each region was used in risk score calculations (Table S1). The migration strategy and location of wintering grounds of each species were also determined [25]. Wintering grounds of migrant species remaining in Europe were identified at a regional (according to the PECBMS regions) rather than country level because data on the precise wintering locations for most breeding populations are not available.

### Validation of the risk assessment framework

#### Components of change to forest habitats

To validate the framework, we assessed a number of land-use and management changes for their impact on food abundance, foraging habitat availability, nesting habitat availability, and nesting success (Table 1, with supporting evidence in Table S3). These changes were identified through an extensive literature search and consultation with a number of ornithological experts specialising in forest systems and representing each of the PECBMS regions included in the analysis. The key impacts of these changes on forest birds were defined in terms of any consequent reduction in the quantity and/or quality of the resources included in the requirements matrix. Forest habitats in Europe were split into three broad types: i) temperate and boreal coniferous dominated forest; ii) temperate and hemi-boreal broadleaf dominated forest; and iii) Mediterranean forest. These categories represent only the first tier of possible forest type classifications but were deemed appropriate for the analyses presented here because of the differences between and similarities within categories in terms of ecological changes that have occurred.

**Table 1:** Major changes to forest habitats identified and their key impacts on forest bird species.

Change to forest habitat	Forest type^1^	Key impacts^2^
1. Increased abundance of small predators	C, B/M	Reduced nest success of non-cavity nesters
2. Increased fire suppression	C	Reduction in invertebrate prey
		Reduction in shrub foraging habitat
		Reduction in early and mid-succession foraging habitat
		Reduction in shrub nesting sites
		Reduction in early and mid-succession nesting habitat
		Reduction in cavity nesting sites
3. Increased grazing pressure	C, B/M, Med	Reduction in shrub foraging habitat
		Reduction in quality of ground foraging habitat
		Reduction in shrub and ground nesting sites
		Reduction in nest success of ground nesters
4. Intensified drainage management	C, B/M	Reduction in below ground and ground dwelling invertebrate prey
		Reduction in shrub foraging sites
		Reduction in shrub nesting sites
5. Intensified soil management	C	Reduction in below ground and ground dwelling invertebrates in early and mid-succession habitat
		Reduction in quality of ground nesting sites in early and mid-succession habitat
6. Intensified thinning	C	Reduction in shrub foraging habitat
		Reduction in shrub nesting habitat
7. Reduced abundance of broadleaf species	C	Reduction in canopy and shrub food resources (invertebrates/seeds/plant material)
		Reduction in shrub and canopy nesting sites
8. Reduced rotation length (including fragmentation effects)	C, B/M	Reduction in old growth foraging habitat
		Reduction in core foraging habitat
		Reduction in old growth succession nesting habitat
		Reduction in core nesting habitat
		Reduction in nesting success in edge habitat
9. Removal of deadwood	C, B/M	Reduction in invertebrate prey
		Reduction in cavity nest sites
10. Reduced area of broadleaf/mixed forest	B/M	Reduction in foraging and nesting habitat
11. Reduction in management	B/M, Med	Reduction in edge foraging habitat
		Reduction in shrub and ground foraging habitat
		Reduction in edge nesting habitat
		Reduction in shrub and ground nesting sites
12. Reduced diversity of tree species	B/M	Reduction in food resources (invertebrates/seeds/plant material)
13. Increased forest fires	Med	Reduction in foraging and nesting habitat
14. Loss to urbanisation	Med	Reduction in foraging and nesting habitat
15. Increased selective logging	Med	Reduction in cavity nests in closed canopy and old growth habitat
		Reduction in cavity nests in closed canopy and old growth habitat

1 Forest type(s) principally affected by changes are indicated: boreal and temperate coniferous dominated (C), hemi-boreal and temperate broadleaf dominated and mixed (B/M) and Mediterranean (Med).

2 Supporting evidence for impacts of changes is provided in Table S3.

#### Risk score calculation

The total risk to individual species as a consequence of the changes listed in Table 1 was quantified by calculating a pan-European risk score for each in three stages. Firstly, we calculated the potential summer and winter risk accrued by each species in each country if it was present there in that season (Stage 1). Individuals of migrant species are not necessarily exposed to the winter risk in the country in which they breed, rather they are exposed to the winter risk in the country or countries in which they over-winter. We therefore calculated the total risk for breeding populations of each species in a given country by combining their potential summer risk in that country with their potential winter risk in the locations where the breeding birds from that country over-winter (Stage 2). Finally, we calculated a pan-European risk score as a weighted average based on relative population size in the constituent countries (Stage 3). The details of each stage are outlined below.

Stage 1: Using the resource requirements matrix, we calculated risk scores associated with each change to forest habitat for each species based on the proportion of the species' resource requirements detrimentally affected by that change and its reliance on forest to provide those resources (see [24] and Appendix S1 for full details). We then calculated potential summer and winter risk for each species in each country by i) summing risk associated with all changes to summer foraging and breeding resources (summer risk) and those to winter foraging resources (winter risk) for each forest change, ii) summing summer and winter risks for all changes occurring in each of the three forest types and iii) calculating a weighted average of summer risk and winter risk based on the relative proportion of each forest type occurring in that country. Potential summer and winter risk scores were calculated separately at this stage to accommodate migration patterns in the calculation of total risk (See Stage 2).

Stage 2: The potential summer risk score calculated for a given species in a given country was assigned as the summer risk accrued by that species in that country if the species was recorded as breeding there. The winter risk accrued by the breeding population of each species in each country was calculated based on migration strategy. For resident populations, the potential winter risk score calculated for the country in which they breed was assigned as the winter risk to which they were exposed. For partially and fully migrant populations, winter risk scores were calculated as the average of potential winter risk in the constituent countries of the region(s) where they over-winter. For those species that winter outside of Europe a winter risk of zero was nominally assigned because we do not have sufficient information to make an informed assessment (see Discussion).

Stage 3: Total risk scores for each species in each country were calculated by summing summer and winter risk. A pan-European risk score was then calculated for each species as the average of its total risk across all countries, weighted by the relative breeding population size in each country [23]. This risk score reflects the detrimental, pan-European impact of past changes to forest habitats on each species and its calculation effectively mirrors the process used by PECBMS to calculate pan-European population trends.

#### Scaling risk

The theory underpinning this risk assessment framework suggests that the level of response of a species to any given habitat change should be dependent on the extent of that change i.e. the response to changes that are more intensive and/or occur over a greater area is expected to be more negative. Given that the scale of each of the changes detailed in Table 1 is likely to differ between countries, we tested the effects of including a scaling mechanism in our risk score calculation process. This adjusted the influence of each change to forest habitat on the final risk score on the basis of the relative extent to which it has occurred in each country. We scaled risk both quantitatively, using pan-European data sources (Table S4), and qualitatively, through consultation with ornithological experts specialising in forest systems (Table S5). When applying quantitative scaling factors, we calculated rates of change over the same time period for which bird population trends were available, or from the closest time period possible where these specific data were not available. Appropriate scaling data were not available for every change listed in Table 1 so surrogates or proxies were employed where necessary. Most commonly, change in timber yield was used to reflect the scale of changes associated with intensification of forestry practices. When applying qualitative scaling factors, data were derived from scores provided by avian/forest ecology experts in each country; these experts were asked to score changes to forest habitats in their country depending on the level of severity, where changes were assessed as not present, minor, moderate, major, or severe (Table S5). Both quantitative and qualitative scaling factors were calculated at a regional scale because data were not available for all countries. Regional rates of change were calculated as the average across constituent countries for which data were available and applied to all countries within each region. Risk scores were recalculated incorporating each type of scaling factor.

#### Testing the relationship between risk and population growth rates

We used General Linear Modelling (GLM) to investigate the relationship between pan-European population trends and pan-European risk score, with separate models for unscaled, quantitatively- and qualitatively-scaled risk. To account for variability in the precision of species' population estimates, models were weighted according to the standard error of the population growth rate estimates. The calculation of total risk assumes that each source of risk has equal weighting in terms of its impact on population growth. This has proven to be a reasonable assumption for farmland biodiversity [20], [22], but we tested whether this also holds for forest systems by decomposing risk in a series of alternative, more complex models that allowed the weighting of different sources of risk to vary. Total risk was decomposed in four ways: by diet and nesting; season; separate forest changes (as in Table 1); and by forest type. Note that these additional models are simply more complex formulations of the model where risk is incorporated in its aggregated form; they do not contain additional independent data, merely the same data partitioned in different ways. In addition, we explored the effects of including migration strategy (resident, short-distance or long-distance) as a categorical predictor in each model. These more complex alternatives were compared to the most parsimonious, total risk model using corrected Akaike's Information Criterion (AICc), with those with AICc values >2 points lower identified as receiving substantially more support, those with AICc values within ±2 having similar support and those with AICc value >2 points higher receiving less support [26]. AICc values for equivalent model structures were also compared across the three scaling mechanisms to assess the relative merits of each approach.

## Results

### Validation of the risk assessment

**Figure 1:**
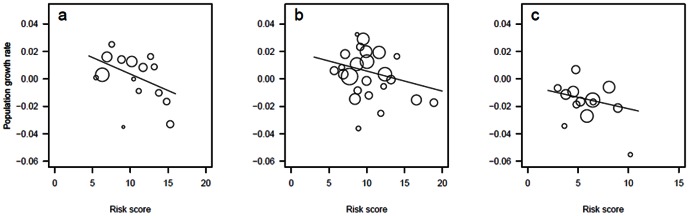
The relationship between risk score (with no scaling mechanism) and annual population growth rate of 52 forest bird species. Species with different migration strategies are presented separately: a) non-migratory (y = −0.002x+0.03, r^2^ = 0.29); b) within Europe migrants (y = −0.001x+0.02, r^2^ = 0.09); c) wintering outside Europe (y = −0.002x+−0.005, r^2^ = 0.07). The sizes of data points are proportional to the standard error of population growth rate estimate, with larger points having smaller standard error and thus greater weight in models. Relationships were tested concurrently with migration strategy as a separate term in the model (P = 0.01, see text for details).

Risk scores derived from assessing the environmental effect of changes to forest habitats across Europe were significantly related to annual population growth rates of forest bird species between 1980 and 2009. When no scaling mechanism was applied, higher risk scores were associated with species with more negative population growth rates and therefore experiencing population declines (Fig 1; F_1,48 = _6.68, P = 0.01), but only when we controlled for migration strategy (P<0.001). There was, however, no significant interaction between risk score and migration strategy (P = 0.83). Risk scores calculated with the quantitative scaling mechanism were not significantly associated with population growth rates, but those calculated with the qualitative scaling mechanism were (F_1,48 = _0.16, P = 0.69 and F_1,48 = _5.84, P = 0.02 respectively).

### Alternative models

Across all scaling mechanisms (no scaling, quantitative and qualitative), models controlling for migration strategy generally received much greater support than those that did not account for it (Table 2). With no scaling mechanism applied and of the models accounting for migration, those with risk decomposed into foraging and nesting components and by season received similar levels of support to the model based on total risk (Δ AICc <±2, Table 2). However, the model accounting for migration and with risk decomposed by forest type received much higher support (Δ AICc  = −12.9).

**Table 2:** Comparison of alternative models describing variation in population growth rate (PGR), assessing the effect of controlling for migration strategy and decomposing risk scores into sub-components by forest type, season, nesting or foraging and individual forest changes.

Model	No scaling		Quantitative scaling		Qualitative scaling	
	AICc	Δ AICc	AICc	Δ AICc	AICc	Δ AICc
PGR∼migration+total risk	−285.5		−278.9		−284.7	
PGR∼migration+coniferous risk+broadleaf risk_+_Mediterranean risk	−298.4	−12.9	−289.4	−10.5	−293.4	−8.7
PGR∼migration+foraging risk +nesting risk	−285.5	0	−280.3	−1.4	−284.3	0.4
PGR∼migration+summer foraging+winter foraging+nesting risk	−284.3	1.2	−278.1	0.8	−288.2	−3.5
PGR∼coniferous risk+broadleaf risk_+_Mediterranean risk	−280.7	4.8	−279.0	−0.1	−278.1	6.6
PGR∼summer foraging+winter foraging+nesting risk	−280.7	4.8	−276.9	2	−278.1	6.6
PGR∼foraging+nesting risk	−277.5	8	−279.3	−0.4	−273.2	11.5
PGR∼total risk	−263.6	21.9	−269.6	9.3	−263.5	21.2
PGR∼migration+change 1 risk+change 2 risk+…+change 22 risk	−236.7	48.8	−243.2	35.7	−252.5	32.2
PGR∼change 1 risk+change 2 risk+…+change 22 risk	−230.2	55.3	−245.8	33.1	−249.2	35.5

Note that model fit was compared between models within the same scaling mechanism and that Δ AICc is calculated as the difference in AICc value from the baseline model of migration plus total risk; this is the most parsimonious formulation of risk score and all other models represent more complex formulations of this rather than containing independent data.

A similar pattern of model support was observed when risk scores were calculated using the qualitative scaling mechanism. Having controlled for migration strategy, models with risk decomposed into foraging and nesting risk received similar support to the total risk model (Δ AICc <2). The model with risk disaggregated by season received slightly more support, and the model with risk decomposed by forest type support received much greater support (Δ AICc  = −8.7; Table 2). For models based on risk calculated using the quantitative scaling mechanism, the model based on migration strategy and forest type again received the greatest support (Δ AICc  = −10.5; Table 2). For this set of models, many more received similar levels of support to the total risk plus migration model, with only the model based on total risk and excluding migration and those based on risk disaggregated by change type (with or without migration) receiving substantially less support (Δ AICc > 9.4).

There was no clear pattern of improvement in fit across model structures by incorporating either the quantitative or qualitative scaling mechanism, with the no scaling version of the migration plus forest type model receiving substantially more support than any other model formulation across all three scaling mechanisms (Table 2). Models with risk scores derived using the qualitative scaling mechanism generally received more support than equivalent models based on risk scores derived using the quantitative mechanism and were more closely aligned to the no scaling models. The qualitative scaling migration plus total risk model received similar levels of support to the equivalent no scaling model but the quantitative scaling version of this model structure received less support (Δ AICc > 2).

## Discussion

### The framework as a conservation tool

The results strongly indicate that population declines in forest birds are causally linked to a loss of resources in forest habitats at a pan-European scale. Impacts of key changes to forest habitats were assessed in terms of which resources are detrimentally affected and how these correspond to the foraging and nesting requirements of each species. Forest habitats are diverse and complex, as are the resource requirements of the species that inhabit them. Despite this, the results demonstrate that even coarse quantification of resource requirements and ecological change can be adequate to assess the risk to which forest birds are exposed following alteration of their habitat. The same trait-based approach has been used to assess risk to various taxa in farmland habitats [20], [21] and this study demonstrates that this approach can be extended to more complex ecosystems, validating its versatility for application in a variety of regions and habitats.

Coarse quantification of risk at a continental scale is useful because it provides a general picture of the health of forest ecosystems, making complex conservation issues more accessible to policy makers, conservation managers and members of the public. Furthermore, whilst conservation and forest management strategies tend to be implemented at a national or local level, they often reflect biodiversity policy and targets set at a Pan-European scale. A continental perspective of forest management is therefore highly relevant and potentially valuable in informing wide scale conservation strategies. Although this present study assesses past risk to forest bird species, the framework lends itself to being used predictively [20], [21], [22] and could thus prove helpful in assessing impacts of not yet implemented changes to forest management, possibly resulting from policy change.

### Loss of resources as a driver of decline

Although there are likely to be multiple drivers of forest bird declines in Europe, our results highlight that resource loss is a key concern. Fragmentation is often cited as possibly the most important threat to forest birds, as it limits dispersal and reduces availability of core habitat [27]. Regardless of landscape structure, however, it is vital that forest habitats contain adequate resources to enable birds to survive and successfully breed. Managed or young forest habitats have been shown to be resource limited in terms of nest sites [28] and food resources [29]. In genuinely undisturbed forest, resource availability is probably of lesser importance; instead nest predation could be fundamental in controlling populations [30]. One of the most obvious resource differences between undisturbed and managed forest is the volume of standing and lying deadwood [31], [32], which is associated with higher invertebrate food and cavity nest resources. This is well recognised and accordingly there are currently efforts to increase the volume of deadwood in forests at a European scale [33].

We provide evidence that resource loss is not only important for resident and short-distance (within Europe) migrants, but also for species wintering outside Europe. Factors acting on passage and on the wintering grounds have long been suspected as being highly significant in the decline of these species [34], raising doubts as to whether European based conservation effort for species wintering outside Europe is worthwhile. Of the species included here, all but one of the species wintering outsides of Europe had a negative population growth rate, whereas less than half of within Europe migrants or resident species did. However, the negative relationship between risk and population growth rate in long distance migrants highlights that together with conservation action at wintering grounds, resource availability within Europe must also be addressed. Migration strategy was an important component of the models in the analysis and including it as a categorical predictor variable better explained the relationship between risk and population growth rate. Since we were unable to quantify the risk that long distance migrants are exposed to on their wintering grounds, these species did not accrue winter risk in our risk assessment. These species therefore had relatively lower risk scores compared to those for which winter risk could be calculated (i.e. resident and short-distance migrants). Short-distance migrant risk scores were, on average, lower than those of resident species while this group also contained a higher proportion of species in decline. It is likely that their risk scores were lower because within Europe migrants accrue much of their risk from Mediterranean forest. Fewer changes to Mediterranean forest were identified compared to the other two forest types and hence risk scores were lower. Although migration was an important predictor, there was not a significant interaction between risk score and migration strategy.

### Limitations of the framework

Taking a broad scale perspective on how changes to forest habitats have affected bird populations inevitably means a compromise with detail, due mainly to data availability and variability in ecological responses. This is apparent in a number of ways within the framework. Firstly, the links between the identified changes to forest habitats and losses of specific resources are not necessarily well established and there is sometimes conflicting evidence (e.g. [35], [36]); in these circumstances a relatively subjective assessment had to be made based on the weight of evidence and expert opinion. Secondly, for species that migrate within Europe, it was not possible to identify over-wintering destinations at a finer resolution than the regional scale. We therefore had to use the average risk across countries within a given region to estimate winter risk for these species, resulting in a loss of information, which may be particularly important if countries within a region have notably differing levels of risk. Likewise, we had to use regional modes for reliance scores in the risk assessment process, as opposed to country-specific scores, because there were only single respondents from some countries. Thirdly, we assumed that species exploit the same resources in different geographical regions although this is known not to be the case for a number of species. For example, Hedge Accentor (*Prunella modularis*) exhibits a strong preference for coniferous forest in some parts of its range and broadleaf in others [25] but we were not able to account for such geographical differences in resource use due to difficulty in ascertaining the boundaries between different areas of preference. More generally, the data on which the framework is reliant tend to be collected and reported within political rather than ecological boundaries even though boundaries defined according to ecological differences may ultimately be more appropriate. The advantage of utilising political boundaries is that policy and management strategies tend to be implemented within these.

Across all three risk scaling mechanisms, the model based on risk scores decomposed by forest type received far greater support than that based on total risk. The improvement in model fit associated with allowing unequal weighting in the effects of risk accrued from each forest type on population dynamics reflects the composite deviation, across species and forest types, from a number of assumptions. Firstly, our risk calculation process makes the necessary but unlikely assumption that if a species is associated with more than one forest type, it will demonstrate equal preference for each and its population distribution across them will be solely dictated by the relative area of each. Secondly, we categorised forests into three types in the interest of practicality and feasibility with regard to data availability, ignoring the substantial differences within these categories. Forest Europe currently recognises 14 distinct forest types which it now endeavours to cover in future forest monitoring [1]. The changes to European forests and their impacts, described in this assessment, will vary across these forest types, as will the bird species assemblages and their responses. Additionally, there are likely to have been important changes to forest habitats that are specific to particular forest types, or to particular regions, that we have not fully accounted for in this analysis. Despite this, the three forest types chosen are linked by common changes, and these in turn were related to the population decline of forest birds. In contrast, allowing risk accrued from different management changes, during different seasons or affecting different resource types did little to improve model fit. This suggests that, across the species considered, the relative influence of these factors on population dynamics can be taken as broadly similar, in line with previous findings in agroecosystems [22]. Furthermore, the total risk model can be considered preferable to any decomposed risk model that received similar support because it is more parsimonious, with the others being more complex formulations that do not include additional independent data [37], [38].

Unscaled risk scores were calculated based on the assumption that changes to forest habitats have occurred uniformly across Europe. This is unrealistic as numerous ecological, economic, political and cultural differences in forest management between countries and regions will cause changes to vary in extent and effect. For example, clear cutting is more common and tends to cover larger areas in Northern countries compared to Central and Eastern Europe. Incorporating the quantitative and qualitative scaling mechanisms was an attempt to account for this variation but they did little to improve model fit and, in the case of the quantitative mechanism, significantly reduced it. Data relating to the extent and intensity of many of the changes assessed in these analyses are not currently available, either at national or pan-European scales. As a consequence, change in timber yield had to be used as a proxy for the extent of many of the changes to forest habitats in the quantitative scaling approach, on the assumption that an increase in yield results from an intensification of management. It is unclear whether this is a reasonable assumption because, unlike the association between agricultural intensification and yield, the association between intensification of forest management and timber yield is not well understood. Monitoring of forest habitats and collation of national scale data across Europe is improving [1], which should aid future investigation into the effects of changing forest habitats, but we emphasise the need for structured and comprehensive collection of data relating to forest management practices across Europe. More importantly, the quantitative scaling mechanism may not have been effective because it was based on the extent of all assessed management changes during the period of bird population monitoring, whereas the extent of some of the assessed changes to forest management occurring before this period may actually be a greater driver of recent population dynamics. Indeed, the time lag between implementation of management practices and their impact on resource availability can be a major obstacle to understanding how forest management is linked to species' population decline. For some management changes assessed, the full impact on resource availability and abundance will be immediately apparent. However, for others, the impacts will increase over time as rotations are realised. Given that forest rotations occur over decades, not uncommonly in excess of a hundred years, these changes could take an equivalent time to have their full impact on population dynamics. Consequently, observed population declines in this study may be driven in part by risk associated with changes to management actions that were implemented some time before the beginning of the population monitoring period. Likewise, populations may not yet have fully responded to management changes recorded during the period studied here. The qualitative scaling may have been more effective than the quantitative approach because, although the experts were asked to consider only the time period covered by the bird monitoring data, their perceptions may have integrated changes that have occurred over a much longer period. It is reassuring to note that disaggregating risk by management change type did not improve model fit for any of the three scaling mechanisms. This implies that risk accrued from each management type, whether its full impact on resource availability is likely to be immediately realised or if it will only be realised over the course of a full rotation, can be taken as having an equal influence on current population dynamics. Time lag effects will be greatest when assessing the impact of management changes at the stand scale and can be expected to decline with increasing spatial scale, as more stands at varying stages in a rotation are incorporated. Our results suggests that, at the spatial and temporal scales considered here, time lag effects are not substantial.

Our risk score calculations only account for the indirect, detrimental impacts of stand-scale changes in forest habitats, as mediated through changes in resource availability. They do not, for example, account for changes in forest habitat driven by factors acting beyond the stand scale. Large scale changes such as climate change are known to be important drivers of forest bird population dynamics [39], but are difficult to associate with the loss of specific resources and thus were not included in the framework. Risk scores also do not account for risk accrued from habitats outside of forests, or forest habitats outside Europe for long distance migrants. Many species occupy both forest and non-forest habitats and may be exposed to additional risk if resources are being lost there too. In addition, as this is a risk rather than an impact assessment framework it does not currently account for any possible benefits of changes in forest habitats. For example, whilst the framework accounts for the detrimental effects of reduced rotation length on resource availability for species associated with old growth forests, it does not account for potential increases in resource availability for species associated with early succession habitats driven by this change. Incorporating these effects would likely improve the explanatory and predictive power of the framework but it is reassuring that we found strong links between risk score and population growth rate, suggesting it captures the main factors driving European forest bird population dynamics.

### Extending the framework

The framework assesses the detrimental effects of past changes on population growth of forest bird species. Demonstrating that there is a likely causal link between changes to forest habitats, their effect on resources and population growth rates means that the framework could be used predictively to assess what effect future changes in forest management may have on birds. The impacts of predicted changes can be scored independently, using the same approach described here, with the derived risk score for each species added to their current risk score to quantify risk in the resultant landscape. Using parameter estimates from the models presented here, population growth rates under the new conditions could then be predicted. Utilising the framework predictively has been successfully achieved at a pan-European scale for farmland birds by assessing the likely effect of further agricultural intensification within Europe [22].

## Conclusions

It is predicted that forest area will continue to increase in Europe [40], although almost all forest is likely to be managed to some extent, either for timber or other human uses such as recreation. Thus, it is critical that we understand the links between management practices, resource availability and biodiversity health if current biodiversity declines are to be halted or reversed, a high priority for nature policy at a European level [41]. We have demonstrated that using a trait-based framework can assist in understanding the causes of decline in European forest birds. Underlying this framework is a simple quantification of resource use and identification of major changes that have occurred in forest habitats. This approach has previously been applied to farmland habitats and this study demonstrates that it can be expanded into other ecosystems, including more complex ones like forests. Understanding the causes of decline associated with past land-use and management changes enables the possible effects of future changes to be predicted. This could contribute to improving the effectiveness and efficiency of conservation actions designed both to mitigate the impacts of past changes and offset the detrimental effects of future changes.

## Supporting Information

Table S1 **Modal reliance scores for European forest birds.**
(DOCX)

Table S2 **Resource requirements matrix for European forest birds.**
(DOCX)

Table S3 **Major changes to European forest habitats in recent decades.**
(DOCX)

Table S4 **Quantitative scaling factors for extent of each forest change.**
(DOCX)

Table S5 **Qualitative scaling factors for extent of each forest change.**
(DOCX)

Appendix S1 **Overview of risk assessment process.**
(DOCX)
