# Supplementary material for: Quantifying the Detrimental Impacts of Land-Use and Management Change on European Forest Bird Populations
Source: PLoS One. 2013 May 21;8(5):e64552. doi: 10.1371/journal.pone.0064552 (PMC3660351; doi:10.1371/journal.pone.0064552)
Supplement: Table S1 — Modal reliance scores for European forest birds. (DOCX) [file pone.0064552.s001.docx]

**Table S1: Modal reliance scores for each species as assessed by ornithological experts^1^ from across four European regions. Numbers in parenthesis are number of respondents from each region. Reliance scores: 1 - Major; 2 – Moderate; 3 – Minor; 0 – Not present in forest**

|  |  | **Regional reliance scores** | | | |
| --- | --- | --- | --- | --- | --- |
| **Common name** | **Taxonomic name** | **East**  (20) | **North**  (7) | **South**  (5) | **West**  (17) |
| Wood Sandpiper | *Tringa Glareola* | 0 | 3 | 0 | 3 |
| Eurasian Sparrowhawk | *Accipiter nisus* | 2 | 1 | 2 | 2 |
| Common Buzzard | *Buteo buteo* | 2 | 2 | 2 | 2 |
| Hazel Grouse | *Bonasa bonasia* | 1 | 1 | 1 | 1 |
| Stock Dove | *Columba oenas* | 1 | 2 | 2 | 2 |
| Common Wood-pigeon | *Columba palumbus* | 2 | 2 | 2 | 2 |
| Common Cuckoo | *Cuculus canorus* | 3 | 2 | 3 | 3 |
| Eurasian Wryneck | *Jynx torquilla* | 3 | 3 | 3 | 2 |
| Eurasian Green Woodpecker | *Picus viridis* | 2 | 2 | 2 | 2 |
| Black Woodpecker | *Dryocopus martius* | 1 | 1 | 1 | 1 |
| Great Spotted Woodpecker | *Dendrocopos major* | 2 | 1 | 2 | 1 |
| Lesser Spotted Woodpecker | *Dendrocopos minor* | 2 | 1 | 2 | 1 |
| Woodlark | *Lullula arborea* | 2 | 3 | 2 | 2 |
| Tree Pipit | *Anthus trivialis* | 2 | 2 | 2 | 2 |
| Winter Wren | *Troglodytes troglodytes* | 2 | 1 | 2 | 2 |
| Hedge Accentor | *Prunella modularis* | 2 | 2 | 3 | 2 |
| European Robin | *Erithacus rubecula* | 2 | 2 | 2 | 2 |
| Common Nightingale | *Luscinia megarhynchos* | 3 | 0 | 2 | 2 |
| Common Redstart | *Phoenicurus phoenicurus* | 2 | 2 | 2 | 2 |
| Eurasian Blackbird | *Turdus merula* | 2 | 2 | 3 | 2 |
| Fieldfare | *Turdus pilaris* | 3 | 2 | 3 | 3 |
| Song Thrush | *Turdus philomelos* | 2 | 1 | 2 | 2 |
| Redwing | *Turdus iliacus* | 2 | 2 | 2 | 3 |
| Mistle Thrush | *Turdus viscivorus* | 1 | 1 | 2 | 2 |
| Icterine Warbler | *Hippolais icterina* | 3 | 1 | 3 | 2 |
| Garden Warbler | *Sylvia borin* | 2 | 1 | 2 | 2 |
| Blackcap | *Sylvia atricapilla* | 2 | 2 | 2 | 2 |
| Wood Warbler | *Phylloscopus sibilatrix* | 1 | 1 | 1 | 1 |
| Common Chiffchaff | *Phylloscopus collybita* | 2 | 1 | 2 | 2 |
| Willow Warbler | *Phylloscopus trochilus* | 2 | 2 | 1 | 2 |
| Goldcrest | *Regulus regulus* | 1 | 1 | 1 | 1 |
| Spotted Flycatcher | *Muscicapa striata* | 2 | 1 | 2 | 2 |
| European Pied Flycatcher | *Ficedula hypoleuca* | 2 | 2 | 1 | 1 |
| Long-tailed Tit | *Aegithalos caudatus* | 2 | 1 | 1 | 2 |
| Marsh Tit | *Poecile palustris* | 2 | 1 | 1 | 1 |
| Willow Tit | *Poecile montanus* | 1 | 1 | 1 | 1 |
| Crested Tit | *Lophophanes cristatus* | 1 | 1 | 1 | 1 |
| Coal Tit | *Periparus ater* | 1 | 1 | 1 | 1 |
| Blue Tit | *Cyanistes caeruleus* | 2 | 2 | 1 | 2 |
| Great Tit | *Parus major* | 2 | 2 | 2 | 2 |
| Wood Nuthatch | *Sitta europaea* | 2 | 0 | 1 | 1 |
| Eurasian Tree-creeper | *Certhia familiaris* | 1 | 1 | 1 | 1 |
| Eurasian Jay | *Garrulus glandarius* | 2 | 1 | 1 | 1 |
| Spotted Nutcracker | *Nucifraga caryocatactes* | 1 | 1 | 1 | 1 |
| Chaffinch | *Fringilla coelebs* | 2 | 2 | 3 | 2 |
| Brambling | *Fringilla montifringilla* | 0 | 1 | 0 | 0 |
| Eurasian Siskin | *Carduelis spinus* | 1 | 1 | 2 | 1 |
| Common Redpoll | *Carduelis flammea* | 3 | 1 | 2 | 2 |
| Eurasian Greenfinch | *Carduelis chloris* | 3 | 3 | 3 | 3 |
| Eurasian Bullfinch | *Pyrrhula pyrrhula* | 1 | 1 | 1 | 2 |
| Hawfinch | *Coccothraustes coccothraustes* | 2 | 3 | 1 | 1 |
| Rustic Bunting | *Emberiza rustica* | 0 | 1 | 0 | 0 |

^1^The following people made contributions as experts: Ainars Aunins, Marc Anton, Ivan Budinski, Tomáš Bělka, Hans-Günther Bauer, Helmut Brücher, Gilles Biver, Sebastian Bugariu, Paul Bellamy, Jakub Čejka, Jaroslav Cepák, Josef Chytil, Dick Coombes, Denis Cachia, Przemysław Chylarecki, Tomasz Chodkiewicz, Elisabeth Charman, Andrew Cristinacce, Jaanus Elts, Mark Eaton, Jiří Flousek, Martin Fejfar, Martin Flade, Marco Gustin, Sergey Golubev, Gennady Grishanov, Igor Gorban, Martin A. Hellicar, David Horal, Henning Heldbjerg, Magne Husby, Süreyya İsfendiyaroğlu, Frédéric Jiguet, Riho Kinks, Zsolt Karcza, Oskars Keišs, Mikhail Kalyakin, Simon Levy, Aleksi Lehikoinen, Csaba Lendvai, Patric Lorgé, Domingos Leitão, Åke Lindström, Kresimir Mikulic, Michael Miltiadou, Riho Marja, Ricardo Martins, Alexander Mischenko, Tomaž Mihelič, Juan Carlos del Moral, John Mallord, Vorobey Nick, Markus Nipkow, Károly Nagy, Vladimir Ostapenko, Jean-Yves Paquet, Derek Pomeroy, Petr Prochazka, Markus Piha, Vasiliy Pchelintsev Jiří Reif, Liutauras Raudonikis, Karel Šťastný, Daróczi J. Szilárd, Irina Borisovna Savinich, Olga Shelomentseva, Sergi Herrando, Sören Svensson, Hans Schmid, Nigel Symes, Norbert Teufelbauer, Juha Tiainen, Rigas Tsiakiris, Chris van Turnhout, Alexandre Vintchevski, Zdeněk Vermouzek, Jari Valkama, Metodija Velevski, Valuev Viktor, Bartłomiej Woźniak, Valery Zakharov, Victor S. Zhukov and Andrei V. Zinoviev.
