# Supplementary material for: Quantifying the Detrimental Impacts of Land-Use and Management Change on European Forest Bird Populations
Source: PLoS One. 2013 May 21;8(5):e64552. doi: 10.1371/journal.pone.0064552 (PMC3660351; doi:10.1371/journal.pone.0064552)
Supplement: Table S2 — Resource requirements matrix for European forest birds. (DOCX) [file pone.0064552.s002.docx]

**Table S2a: Resource matrix (i): summer diet and foraging resource use for the 52 species used in the analysis.**

|  |  | |  | |  | |  | |  |  |  | |  | |  |  |  |  |  |  |  |  |
| --- | --- | --- | --- | --- | --- | --- | --- | --- | --- | --- | --- | --- | --- | --- | --- | --- | --- | --- | --- | --- | --- | --- |
|  | Diet | | | | | | | | |  | Forest type | | | | |  | Horizontal habitat | |  | Vertical habitat | | |
| Species | Below-ground inverts | Above-ground inverts | | Plant material | | Seeds | | Vertebrates | |  | Deciduous | Conifer | | Mixed | |  | Edge | Core |  | Ground | Shrub | Canopy |
| *Tringa Glareola* | *0* | 1 | | 0 | | 0 | | 0 | |  | 0 | 1 | | 0 | |  | 1 | 0 |  | 1 | 0 | 0 |
| *Accipiter nisus* | 0 | 0 | | 0 | | 0 | | 1 | |  | 0 | 1 | | 1 | |  | 1 | 1 |  | 0 | 1 | 1 |
| *Buteo buteo* | 1 | 1 | | 0 | | 0 | | 1 | |  | 1 | 0 | | 0 | |  | 1 | 0 |  | 1 | 0 | 0 |
| *Bonasa bonasia* | 0 | 0 | | 1 | | 1 | | 0 | |  | 0 | 1 | | 1 | |  | 1 | 0 |  | 1 | 0 | 0 |
| *Columba oenas* | 0 | 0 | | 1 | | 1 | | 0 | |  | 1 | 1 | | 1 | |  | 1 | 0 |  | 1 | 0 | 0 |
| *Columba palumbus* | 0 | 0 | | 1 | | 1 | | 0 | |  | 1 | 1 | | 1 | |  | 1 | 0 |  | 1 | 1 | 1 |
| *Cuculus canorus* | 0 | 1 | | 0 | | 0 | | 0 | |  | 1 | 1 | | 1 | |  | 1 | 1 |  | 1 | 0 | 0 |
| *Jynx torquilla* | 0 | 1 | | 0 | | 0 | | 0 | |  | 1 | 0 | | 1 | |  | 1 | 0 |  | 1 | 0 | 0 |
| *Picus viridis* | 0 | 1 | | 0 | | 0 | | 0 | |  | 1 | 0 | | 1 | |  | 1 | 0 |  | 1 | 0 | 0 |
| *Dryocopus martius* | 0 | 1 | | 0 | | 0 | | 0 | |  | 1 | 1 | | 1 | |  | 0 | 1 |  | 1 | 0 | 1 |
| *Dendrocopos major* | 0 | 1 | | 0 | | 0 | | 0 | |  | 1 | 1 | | 1 | |  | 1 | 1 |  | 0 | 0 | 1 |
| *Dendrocopos minor* | 0 | 1 | | 0 | | 0 | | 0 | |  | 1 | 0 | | 1 | |  | 1 | 0 |  | 0 | 1 | 1 |
| *Lullula arborea* | 0 | 1 | | 0 | | 0 | | 0 | |  | 1 | 1 | | 1 | |  | 1 | 0 |  | 1 | 0 | 0 |
| *Anthus trivialis* | 0 | 1 | | 0 | | 0 | | 0 | |  | 1 | 1 | | 1 | |  | 1 | 0 |  | 1 | 1 | 0 |
| *Troglodytes troglodytes* | 0 | 1 | | 0 | | 0 | | 0 | |  | 1 | 1 | | 1 | |  | 1 | 1 |  | 1 | 1 | 0 |
| *Prunella modularis* | 0 | 1 | | 0 | | 0 | | 0 | |  | 1 | 1 | | 1 | |  | 1 | 0 |  | 1 | 1 | 0 |
| *Erithacus rubecula* | 0 | 1 | | 0 | | 0 | | 0 | |  | 1 | 1 | | 1 | |  | 1 | 0 |  | 1 | 0 | 0 |
| *Luscinia megarhynchos* | 0 | 1 | | 0 | | 0 | | 0 | |  | 1 | 0 | | 0 | |  | 1 | 0 |  | 1 | 1 | 0 |
| *Phoenicurus phoenicurus* | 0 | 1 | | 1 | | 0 | | 0 | |  | 1 | 0 | | 1 | |  | 1 | 0 |  | 1 | 1 | 1 |
| *Turdus merula* | 1 | 1 | | 0 | | 0 | | 0 | |  | 1 | 1 | | 1 | |  | 1 | 1 |  | 1 | 1 | 0 |
| *Turdus pilaris* | 1 | 1 | | 1 | | 0 | | 0 | |  | 1 | 1 | | 1 | |  | 1 | 0 |  | 1 | 1 | 1 |
| *Turdus philomelos* | 1 | 1 | | 1 | | 0 | | 0 | |  | 1 | 1 | | 1 | |  | 1 | 0 |  | 1 | 1 | 0 |
| *Turdus iliacus* | 1 | 1 | | 0 | | 0 | | 0 | |  | 1 | 0 | | 1 | |  | 1 | 0 |  | 1 | 1 | 1 |
| *Turdus viscivorus* | 1 | 1 | | 0 | | 0 | | 0 | |  | 1 | 1 | | 1 | |  | 1 | 0 |  | 1 | 1 | 1 |
| *Hippolais icterina* | 0 | 1 | | 1 | | 0 | | 0 | |  | 1 | 0 | | 1 | |  | 1 | 0 |  | 0 | 1 | 1 |
| *Sylvia borin* | 0 | 1 | | 0 | | 0 | | 0 | |  | 1 | 0 | | 1 | |  | 1 | 0 |  | 1 | 1 | 0 |
| *Sylvia atricapilla* | 0 | 1 | | 0 | | 0 | | 0 | |  | 1 | 0 | | 1 | |  | 1 | 1 |  | 0 | 1 | 1 |
| *Phylloscopus sibilatrix* | 0 | 1 | | 0 | | 0 | | 0 | |  | 1 | 1 | | 1 | |  | 0 | 1 |  | 0 | 0 | 1 |
| *Phylloscopus collybita* | 0 | 1 | | 0 | | 0 | | 0 | |  | 1 | 1 | | 1 | |  | 1 | 0 |  | 0 | 1 | 1 |
| *Phylloscopus trochilus* | 0 | 1 | | 0 | | 0 | | 0 | |  | 1 | 1 | | 1 | |  | 1 | 0 |  | 0 | 1 | 1 |
| *Regulus regulus* | 0 | 1 | | 0 | | 0 | | 0 | |  | 0 | 1 | | 0 | |  | 1 | 1 |  | 0 | 0 | 1 |
| *Muscicapa striata* | 0 | 1 | | 0 | | 0 | | 0 | |  | 1 | 0 | | 1 | |  | 1 | 0 |  | 0 | 0 | 1 |
| *Ficedula hypoleuca* | 0 | 1 | | 0 | | 0 | | 0 | |  | 1 | 0 | | 1 | |  | 1 | 1 |  | 0 | 0 | 1 |
| *Aegithalos caudatus* | 0 | 1 | | 0 | | 0 | | 0 | |  | 1 | 1 | | 1 | |  | 1 | 1 |  | 0 | 1 | 1 |
| *Poecile palustris* | 0 | 1 | | 0 | | 0 | | 0 | |  | 1 | 0 | | 1 | |  | 1 | 1 |  | 1 | 1 | 0 |
| *Poecile montanus* | 0 | 1 | | 0 | | 0 | | 0 | |  | 1 | 1 | | 1 | |  | 1 | 1 |  | 0 | 1 | 1 |
| *Lophophanes cristatus* | 0 | 1 | | 0 | | 0 | | 0 | |  | 0 | 1 | | 0 | |  | 0 | 1 |  | 1 | 1 | 1 |
| *Periparus ater* | 0 | 1 | | 0 | | 0 | | 0 | |  | 0 | 1 | | 0 | |  | 1 | 1 |  | 0 | 0 | 1 |
| *Cyanistes caeruleus* | 0 | 1 | | 0 | | 0 | | 0 | |  | 1 | 0 | | 0 | |  | 1 | 1 |  | 0 | 1 | 1 |
| *Parus major* | 0 | 1 | | 0 | | 0 | | 0 | |  | 1 | 0 | | 1 | |  | 1 | 1 |  | 0 | 0 | 1 |
| *Sitta europaea* | 0 | 1 | | 0 | | 0 | | 0 | |  | 1 | 0 | | 1 | |  | 1 | 1 |  | 0 | 0 | 1 |
| *Certhia familiaris* | 0 | 1 | | 0 | | 0 | | 0 | |  | 0 | 1 | | 1 | |  | 1 | 1 |  | 0 | 0 | 1 |
| *Garrulus glandarius* | 0 | 1 | | 1 | | 1 | | 0 | |  | 1 | 0 | | 1 | |  | 0 | 1 |  | 0 | 0 | 1 |
| *Nucifraga caryocatactes* | 0 | 1 | | 1 | | 1 | | 0 | |  | 0 | 1 | | 0 | |  | 1 | 1 |  | 1 | 1 | 1 |
| *Fringilla coelebs* | 0 | 1 | | 0 | | 0 | | 0 | |  | 1 | 1 | | 1 | |  | 1 | 1 |  | 0 | 1 | 1 |
| *Fringilla montifringilla* | 0 | 1 | | 1 | | 1 | | 0 | |  | 1 | 1 | | 1 | |  | 1 | 0 |  | 0 | 1 | 1 |
| *Carduelis spinus* | 0 | 1 | | 0 | | 1 | | 0 | |  | 0 | 1 | | 1 | |  | 1 | 1 |  | 0 | 0 | 1 |
| *Carduelis flammea* | 0 | 1 | | 0 | | 1 | | 0 | |  | 1 | 1 | | 1 | |  | 1 | 0 |  | 1 | 0 | 1 |
| *Carduelis chloris* | 0 | 0 | | 0 | | 1 | | 0 | |  | 1 | 1 | | 1 | |  | 1 | 0 |  | 1 | 1 | 1 |
| *Pyrrhula pyrrhula* | 0 | 0 | | 1 | | 1 | | 0 | |  | 1 | 1 | | 1 | |  | 1 | 0 |  | 0 | 1 | 0 |
| *Coccothraustes coccothraustes* | 0 | 1 | | 1 | | 1 | | 0 | |  | 1 | 0 | | 1 | |  | 1 | 1 |  | 0 | 0 | 1 |
| *Emberiza rustica* | 0 | 1 | | 0 | | 1 | | 0 | |  | 1 | 1 | | 1 | |  | 1 | 0 |  | 1 | 0 | 0 |

**Table S2b: Resource matrix (ii): winter diet and foraging resource use for the 52 species used in the analysis.**

|  | Diet | | | | |  | Forest type | | |  | Horizontal habitat | |  | Vertical habitat | | |
| --- | --- | --- | --- | --- | --- | --- | --- | --- | --- | --- | --- | --- | --- | --- | --- | --- |
| Species | Below-ground inverts | Above-ground inverts | Plant material | Seeds | Vertebrates |  | Deciduous | Conifer | Mixed |  | Edge | Core |  | Ground | Shrub | Canopy |
| *Tringa Glareola* | 0 | 0 | 0 | 0 | 0 |  | 0 | 0 | 0 |  | 0 | 0 |  | 0 | 0 | 0 |
| *Accipiter nisus* | 0 | 0 | 0 | 0 | 1 |  | 0 | 1 | 1 |  | 1 | 1 |  | 0 | 1 | 1 |
| *Buteo buteo* | 1 | 1 | 0 | 0 | 1 |  | 1 | 0 | 0 |  | 1 | 0 |  | 1 | 0 | 0 |
| *Bonasa bonasia* | 0 | 0 | 1 | 1 | 0 |  | 0 | 1 | 1 |  | 1 | 0 |  | 0 | 1 | 1 |
| *Columba oenas* | 0 | 0 | 1 | 1 | 0 |  | 1 | 1 | 1 |  | 1 | 0 |  | 1 | 0 | 0 |
| *Columba palumbus* | 0 | 0 | 1 | 1 | 0 |  | 1 | 1 | 1 |  | 1 | 0 |  | 1 | 1 | 1 |
| *Cuculus canorus* | 0 | 0 | 0 | 0 | 0 |  | 0 | 0 | 0 |  | 0 | 0 |  | 0 | 0 | 0 |
| *Jynx torquilla* | 0 | 0 | 0 | 0 | 0 |  | 0 | 0 | 0 |  | 0 | 0 |  | 0 | 0 | 0 |
| *Picus viridis* | 1 | 1 | 0 | 0 | 0 |  | 1 | 0 | 1 |  | 1 | 0 |  | 1 | 0 | 0 |
| *Dryocopus martius* | 0 | 1 | 0 | 0 | 0 |  | 1 | 1 | 1 |  | 1 | 1 |  | 1 | 0 | 1 |
| *Dendrocopos major* | 0 | 1 | 0 | 1 | 0 |  | 1 | 1 | 1 |  | 1 | 1 |  | 0 | 0 | 1 |
| *Dendrocopos minor* | 0 | 1 | 0 | 0 | 0 |  | 1 | 0 | 1 |  | 1 | 0 |  | 0 | 1 | 1 |
| *Lullula arborea* | 0 | 0 | 0 | 1 | 0 |  | 1 | 1 | 1 |  | 1 | 0 |  | 1 | 1 | 0 |
| *Anthus trivialis* | 0 | 0 | 0 | 0 | 0 |  | 0 | 0 | 0 |  | 0 | 0 |  | 0 | 0 | 0 |
| *Troglodytes troglodytes* | 0 | 1 | 0 | 0 | 0 |  | 1 | 1 | 1 |  | 1 | 1 |  | 1 | 1 | 0 |
| *Prunella modularis* | 0 | 1 | 0 | 1 | 0 |  | 1 | 1 | 1 |  | 1 | 0 |  | 1 | 1 | 0 |
| *Erithacus rubecula* | 0 | 1 | 1 | 1 | 0 |  | 1 | 1 | 1 |  | 1 | 0 |  | 1 | 0 | 0 |
| *Luscinia megarhynchos* | 0 | 0 | 0 | 0 | 0 |  | 0 | 0 | 0 |  | 0 | 0 |  | 0 | 0 | 0 |
| *Phoenicurus phoenicurus* | 0 | 0 | 0 | 0 | 0 |  | 0 | 0 | 0 |  | 0 | 0 |  | 0 | 0 | 0 |
| *Turdus merula* | 1 | 1 | 1 | 0 | 0 |  | 1 | 1 | 1 |  | 1 | 1 |  | 1 | 1 | 0 |
| *Turdus pilaris* | 1 | 1 | 1 | 0 | 0 |  | 1 | 1 | 1 |  | 1 | 0 |  | 1 | 1 | 1 |
| *Turdus philomelos* | 1 | 1 | 1 | 0 | 0 |  | 1 | 1 | 1 |  | 1 | 0 |  | 1 | 1 | 0 |
| *Turdus iliacus* | 1 | 1 | 1 | 0 | 0 |  | 1 | 0 | 1 |  | 1 | 0 |  | 1 | 1 | 1 |
| *Turdus viscivorus* | 1 | 1 | 1 | 0 | 0 |  | 1 | 1 | 1 |  | 1 | 0 |  | 1 | 1 | 1 |
| *Hippolais icterina* | 0 | 0 | 0 | 0 | 0 |  | 0 | 0 | 0 |  | 0 | 0 |  | 0 | 0 | 0 |
| *Sylvia borin* | 0 | 0 | 0 | 0 | 0 |  | 0 | 0 | 0 |  | 0 | 0 |  | 0 | 0 | 0 |
| *Sylvia atricapilla* | 0 | 0 | 1 | 0 | 0 |  | 1 | 0 | 1 |  | 1 | 1 |  | 1 | 1 | 1 |
| *Phylloscopus sibilatrix* | 0 | 0 | 0 | 0 | 0 |  | 0 | 0 | 0 |  | 0 | 0 |  | 0 | 0 | 0 |
| *Phylloscopus collybita* | 0 | 1 | 1 | 0 | 0 |  | 1 | 1 | 1 |  | 1 | 0 |  | 0 | 1 | 1 |
| *Phylloscopus trochilus* | 0 | 0 | 0 | 0 | 0 |  | 0 | 0 | 0 |  | 0 | 0 |  | 0 | 0 | 0 |
| *Regulus regulus* | 0 | 1 | 0 | 0 | 0 |  | 1 | 1 | 1 |  | 1 | 1 |  | 0 | 1 | 1 |
| *Muscicapa striata* | 0 | 0 | 0 | 0 | 0 |  | 0 | 0 | 0 |  | 0 | 0 |  | 0 | 0 | 0 |
| *Ficedula hypoleuca* | 0 | 0 | 0 | 0 | 0 |  | 0 | 0 | 0 |  | 0 | 0 |  | 0 | 0 | 0 |
| *Aegithalos caudatus* | 0 | 1 | 0 | 0 | 0 |  | 1 | 0 | 1 |  | 1 | 1 |  | 0 | 1 | 1 |
| *Poecile palustris* | 0 | 1 | 1 | 1 | 0 |  | 1 | 0 | 1 |  | 1 | 1 |  | 1 | 1 | 1 |
| *Poecile montanus* | 0 | 0 | 0 | 1 | 0 |  | 1 | 1 | 1 |  | 1 | 1 |  | 0 | 1 | 1 |
| *Lophophanes cristatus* | 0 | 1 | 1 | 1 | 0 |  | 0 | 1 | 0 |  | 0 | 1 |  | 1 | 1 | 1 |
| *Periparus ater* | 0 | 1 | 0 | 1 | 0 |  | 0 | 1 | 0 |  | 1 | 1 |  | 0 | 0 | 1 |
| *Cyanistes caeruleus* | 0 | 1 | 1 | 1 | 0 |  | 1 | 0 | 0 |  | 1 | 1 |  | 0 | 1 | 1 |
| *Parus major* | 0 | 1 | 1 | 1 | 0 |  | 1 | 0 | 1 |  | 1 | 1 |  | 1 | 1 | 1 |
| *Sitta europaea* | 0 | 1 | 0 | 1 | 0 |  | 1 | 0 | 1 |  | 1 | 1 |  | 0 | 0 | 1 |
| *Certhia familiaris* | 0 | 1 | 0 | 0 | 0 |  | 0 | 1 | 1 |  | 1 | 1 |  | 0 | 0 | 1 |
| *Garrulus glandarius* | 0 | 1 | 1 | 1 | 0 |  | 1 | 0 | 1 |  | 0 | 1 |  | 1 | 0 | 1 |
| *Nucifraga caryocatactes* | 0 | 0 | 0 | 1 | 0 |  | 0 | 1 | 0 |  | 1 | 1 |  | 1 | 1 | 1 |
| *Fringilla coelebs* | 0 | 0 | 1 | 1 | 0 |  | 1 | 1 | 1 |  | 1 | 1 |  | 1 | 0 | 0 |
| *Fringilla montifringilla* | 0 | 0 | 1 | 1 | 0 |  | 1 | 1 | 1 |  | 1 | 0 |  | 1 | 0 | 0 |
| *Carduelis spinus* | 0 | 0 | 0 | 1 | 0 |  | 1 | 1 | 1 |  | 1 | 1 |  | 0 | 0 | 1 |
| *Carduelis flammea* | 0 | 0 | 0 | 1 | 0 |  | 1 | 1 | 1 |  | 1 | 0 |  | 1 | 0 | 1 |
| *Carduelis chloris* | 0 | 0 | 0 | 1 | 0 |  | 1 | 1 | 1 |  | 1 | 0 |  | 1 | 1 | 1 |
| *Pyrrhula pyrrhula* | 0 | 0 | 1 | 1 | 0 |  | 1 | 1 | 1 |  | 1 | 0 |  | 0 | 1 | 0 |
| *Coccothraustes coccothraustes* | 0 | 0 | 1 | 1 | 0 |  | 1 | 0 | 1 |  | 1 | 1 |  | 1 | 1 | 1 |
| *Emberiza rustica* | 0 | 0 | 0 | 0 | 0 |  | 0 | 0 | 0 |  | 0 | 0 |  | 0 | 0 | 0 |

**Table S2c: Resource matrix (iii): nesting resource use for the 52 species used in the analysis.**

|  | Nest type | | |  | Forest type | | |  | Horizontal habitat | |  | Vertical habitat | | |
| --- | --- | --- | --- | --- | --- | --- | --- | --- | --- | --- | --- | --- | --- | --- |
| Species | Hole - Dead wood | Hole - live wood | External |  | Deciduous | Conifer | Mixed |  | Edge | Core |  | Ground | Shrub | Canopy |
| *Tringa Glareola* | 0 | 0 | 1 |  | 0 | 1 | 0 |  | 1 | 0 |  | 1 | 0 | 0 |
| *Accipiter nisus* | 0 | 0 | 1 |  | 1 | 1 | 1 |  | 1 | 1 |  | 0 | 0 | 1 |
| *Buteo buteo* | 0 | 0 | 1 |  | 1 | 0 | 0 |  | 1 | 0 |  | 0 | 0 | 1 |
| *Bonasa bonasia* | 0 | 0 | 1 |  | 0 | 1 | 1 |  | 0 | 1 |  | 1 | 0 | 0 |
| *Columba oenas* | 1 | 0 | 0 |  | 1 | 1 | 1 |  | 1 | 0 |  | 0 | 0 | 1 |
| *Columba palumbus* | 0 | 0 | 1 |  | 1 | 1 | 1 |  | 1 | 0 |  | 0 | 0 | 1 |
| *Cuculus canorus* | 1 | 0 | 1 |  | 1 | 1 | 1 |  | 1 | 1 |  | 1 | 1 | 1 |
| *Jynx torquilla* | 1 | 0 | 0 |  | 1 | 0 | 1 |  | 1 | 0 |  | 0 | 0 | 1 |
| *Picus viridis* | 1 | 1 | 0 |  | 1 | 0 | 1 |  | 1 | 0 |  | 0 | 0 | 1 |
| *Dryocopus martius* | 1 | 1 | 0 |  | 1 | 1 | 1 |  | 0 | 1 |  | 0 | 0 | 1 |
| *Dendrocopos major* | 1 | 1 | 0 |  | 1 | 1 | 1 |  | 1 | 1 |  | 0 | 0 | 1 |
| *Dendrocopos minor* | 1 | 0 | 0 |  | 1 | 0 | 1 |  | 1 | 0 |  | 0 | 0 | 1 |
| *Lullula arborea* | 0 | 0 | 1 |  | 1 | 1 | 1 |  | 1 | 0 |  | 1 | 0 | 0 |
| *Anthus trivialis* | 0 | 0 | 1 |  | 1 | 1 | 1 |  | 1 | 0 |  | 1 | 0 | 0 |
| *Troglodytes troglodytes* | 1 | 0 | 1 |  | 1 | 1 | 1 |  | 1 | 1 |  | 1 | 1 | 1 |
| *Prunella modularis* | 0 | 0 | 1 |  | 1 | 1 | 1 |  | 1 | 0 |  | 0 | 1 | 0 |
| *Erithacus rubecula* | 1 | 0 | 1 |  | 1 | 1 | 1 |  | 1 | 0 |  | 0 | 1 | 0 |
| *Luscinia megarhynchos* | 0 | 0 | 1 |  | 1 | 0 | 0 |  | 1 | 0 |  | 1 | 1 | 0 |
| *Phoenicurus phoenicurus* | 1 | 0 | 1 |  | 1 | 0 | 1 |  | 1 | 0 |  | 0 | 0 | 1 |
| *Turdus merula* | 0 | 0 | 1 |  | 1 | 1 | 1 |  | 1 | 1 |  | 0 | 1 | 0 |
| *Turdus pilaris* | 0 | 0 | 1 |  | 1 | 1 | 1 |  | 1 | 0 |  | 0 | 0 | 1 |
| *Turdus philomelos* | 0 | 0 | 1 |  | 1 | 1 | 1 |  | 1 | 0 |  | 0 | 1 | 0 |
| *Turdus iliacus* | 0 | 0 | 1 |  | 1 | 0 | 1 |  | 1 | 0 |  | 1 | 1 | 0 |
| *Turdus viscivorus* | 0 | 0 | 1 |  | 1 | 1 | 1 |  | 1 | 0 |  | 0 | 0 | 1 |
| *Hippolais icterina* | 0 | 0 | 1 |  | 1 | 0 | 1 |  | 1 | 0 |  | 0 | 1 | 1 |
| *Sylvia borin* | 0 | 0 | 1 |  | 1 | 0 | 1 |  | 1 | 0 |  | 0 | 1 | 0 |
| *Sylvia atricapilla* | 0 | 0 | 1 |  | 1 | 0 | 1 |  | 1 | 1 |  | 0 | 1 | 0 |
| *Phylloscopus sibilatrix* | 0 | 0 | 1 |  | 1 | 1 | 1 |  | 0 | 1 |  | 1 | 0 | 0 |
| *Phylloscopus collybita* | 0 | 0 | 1 |  | 1 | 1 | 1 |  | 1 | 0 |  | 1 | 1 | 0 |
| *Phylloscopus trochilus* | 0 | 0 | 1 |  | 1 | 1 | 1 |  | 1 | 0 |  | 1 | 1 | 0 |
| *Regulus regulus* | 0 | 0 | 1 |  | 0 | 1 | 0 |  | 1 | 1 |  | 0 | 0 | 1 |
| *Muscicapa striata* | 0 | 0 | 1 |  | 1 | 0 | 1 |  | 1 | 0 |  | 0 | 0 | 1 |
| *Ficedula hypoleuca* | 1 | 0 | 0 |  | 1 | 0 | 1 |  | 1 | 1 |  | 0 | 0 | 1 |
| *Aegithalos caudatus* | 0 | 0 | 1 |  | 1 | 0 | 1 |  | 1 | 0 |  | 0 | 1 | 0 |
| *Poecile palustris* | 1 | 0 | 0 |  | 1 | 0 | 1 |  | 1 | 1 |  | 1 | 1 | 1 |
| *Poecile montanus* | 1 | 0 | 0 |  | 1 | 1 | 1 |  | 1 | 1 |  | 1 | 0 | 0 |
| *Lophophanes cristatus* | 1 | 0 | 0 |  | 0 | 1 | 0 |  | 0 | 1 |  | 1 | 0 | 1 |
| *Periparus ater* | 1 | 0 | 0 |  | 0 | 1 | 0 |  | 1 | 1 |  | 1 | 0 | 1 |
| *Cyanistes caeruleus* | 1 | 0 | 0 |  | 1 | 0 | 0 |  | 1 | 1 |  | 0 | 0 | 1 |
| *Parus major* | 1 | 0 | 0 |  | 1 | 0 | 1 |  | 1 | 1 |  | 0 | 0 | 1 |
| *Sitta europaea* | 1 | 0 | 0 |  | 1 | 0 | 1 |  | 0 | 1 |  | 0 | 0 | 1 |
| *Certhia familiaris* | 1 | 0 | 1 |  | 1 | 1 | 1 |  | 1 | 1 |  | 0 | 0 | 1 |
| *Garrulus glandarius* | 0 | 0 | 1 |  | 1 | 0 | 1 |  | 0 | 1 |  | 0 | 1 | 0 |
| *Nucifraga caryocatactes* | 0 | 0 | 1 |  | 0 | 1 | 0 |  | 0 | 1 |  | 0 | 0 | 1 |
| *Fringilla coelebs* | 0 | 0 | 1 |  | 1 | 1 | 1 |  | 1 | 1 |  | 0 | 1 | 1 |
| *Fringilla montifringilla* | 0 | 0 | 1 |  | 1 | 1 | 1 |  | 1 | 1 |  | 0 | 0 | 1 |
| *Carduelis spinus* | 0 | 0 | 1 |  | 0 | 1 | 1 |  | 1 | 1 |  | 0 | 0 | 1 |
| *Carduelis flammea* | 0 | 0 | 1 |  | 1 | 1 | 1 |  | 1 | 0 |  | 0 | 1 | 1 |
| *Carduelis chloris* | 0 | 0 | 1 |  | 1 | 1 | 1 |  | 1 | 0 |  | 0 | 1 | 0 |
| *Pyrrhula pyrrhula* | 0 | 0 | 1 |  | 1 | 1 | 1 |  | 1 | 0 |  | 0 | 1 | 0 |
| *Coccothraustes coccothraustes* | 0 | 0 | 1 |  | 1 | 0 | 1 |  | 1 | 1 |  | 0 | 0 | 1 |
| *Emberiza rustica* | 0 | 0 | 1 |  | 1 | 1 | 1 |  | 1 | 0 |  | 1 | 0 | 0 |
