# Supplementary material for: Quantifying the Detrimental Impacts of Land-Use and Management Change on European Forest Bird Populations
Source: PLoS One. 2013 May 21;8(5):e64552. doi: 10.1371/journal.pone.0064552 (PMC3660351; doi:10.1371/journal.pone.0064552)
Supplement: Table S3 — Major changes to European forest habitats in recent decades. (DOCX) [file pone.0064552.s003.docx]

**Table S3: Major changes to forest habitats identified and their key impacts on forest bird species, with supporting references.**

| **Change to forest habitat** | **Key impacts** | **References** |
| --- | --- | --- |
| 1. Increased abundance of small predators | - Reduced nest success of non-cavity nesters | [[1](#_ENREF_1)] |
| 2. Increased fire suppression | - Reduction in invertebrate prey - Reduction in shrub foraging habitat - Reduction in early and mid-succession foraging habitat - Reduction in shrub nesting sites - Reduction in early and mid-succession nesting habitat - Reduction in cavity nesting sites | [[2](#_ENREF_2),[3](#_ENREF_3)]  [[1](#_ENREF_1)]  [[4](#_ENREF_4)]  [[1](#_ENREF_1),[5](#_ENREF_5)]  [[4](#_ENREF_4),[5](#_ENREF_5)]  [[2](#_ENREF_2),[5](#_ENREF_5)] |
| 3. Increased grazing pressure from domestic and wild herbivores | - Reduction in shrub foraging habitat - Reduction in quality of ground foraging habitat - Reduction in shrub and ground nesting sites - Reduction in nest success of ground nesters | [[6](#_ENREF_6),[7](#_ENREF_7)]  [[6](#_ENREF_6),[7](#_ENREF_7)]  [[6](#_ENREF_6),[7](#_ENREF_7)]  [[6](#_ENREF_6)] |
| 4. Intensified drainage management | - Reduction in below ground and ground dwelling invertebrate prey - Reduction in shrub foraging sites - Reduction in shrub nesting sites | [[8](#_ENREF_8),[9](#_ENREF_9)]  [[10](#_ENREF_10),[11](#_ENREF_11)]  [[10](#_ENREF_10),[11](#_ENREF_11)] |
| 5. Intensified soil management | - Reduction in below ground and ground dwelling invertebrates in early and mid-succession habitat - Reduction in quality of ground nesting sites in early and mid-succession habitat | [[5](#_ENREF_5),[12](#_ENREF_12)]  [[12](#_ENREF_12)] |
| 6. Intensified thinning | - Reduction in shrub foraging habitat - Reduction in shrub nesting habitat | [[13](#_ENREF_13)]  [18] |
| 7. Reduced abundance of broadleaf species | - Reduction in canopy and shrub food resources (invertebrates/seeds/plant material) - Reduction in shrub and canopy nesting sites | [[14](#_ENREF_14),[15](#_ENREF_15)]  [[16](#_ENREF_16),[17](#_ENREF_17)] |
| 8. Reduced rotation length (including fragmentation effects) | - Reduction in old growth foraging habitat - Reduction in core foraging habitat - Reduction in old growth succession nesting habitat - Reduction in core nesting habitat - Reduction in nesting success in edge habitat | [[18](#_ENREF_18),[19](#_ENREF_19)]  [[20](#_ENREF_20)]  [[18](#_ENREF_18),[19](#_ENREF_19)]  [[20](#_ENREF_20)]  [[1](#_ENREF_1),[21](#_ENREF_21)] |
| 9. Removal of deadwood | - Reduction in invertebrate prey - Reduction in cavity nest sites | [[5](#_ENREF_5)]  [[22](#_ENREF_22)] |
| 10. Reduced area of broadleaf/mixed forest | - Reduction in broadleaf and mixed forest foraging and nesting habitat | By definition |
| 11. Reduction in management | - Reduction in edge foraging habitat - Reduction in shrub and ground foraging habitat - Reduction in edge nesting habitat - Reduction in shrub and ground nesting sites | [[23](#_ENREF_23)]  [[2](#_ENREF_2),[23](#_ENREF_23)]  [[23](#_ENREF_23)]  [[2](#_ENREF_2),[23](#_ENREF_23)] |
| 12. Reduced diversity of tree species | - Reduction in food resources (invertebrates/seeds/plant material) | [[15](#_ENREF_15),[17](#_ENREF_17)] |
| 13. Increased forest fires | - Reduction in Mediterranean foraging and nesting habitat | [[24](#_ENREF_24)] |
| 14. Loss of habitat through urbanisation | - Reduction in Mediterranean foraging and nesting habitat | By definition |
| 15. Increased selective logging | - Reduction in invertebrates in closed canopy and old growth habitat - Reduction in cavity nests in closed canopy and old growth habitat | [[25](#_ENREF_25)]  [[25](#_ENREF_25)] |

**References**

1. Brawn JD, Robinson SK, Thompson III FR (2001) The role of disturbance in the ecology and conservation of birds. Annual Review of Ecology and Systematics: 251-276.

2. Kalies EL, Chambers CL, Covington WW (2010) Wildlife responses to thinning and burning treatments in southwestern conifer forests: A meta-analysis. Forest Ecology and Management 259: 333-342.

3. Hutto RL (1995) Composition of bird communities following stand replacement fires in northern Rocky Mountain (USA) conifer forests. Conservation Biology 9: 1041-1058.

4. Betts MG, Hagar JC, Rivers JW, Alexander JD, McGarigal K, et al. (2010) Thresholds in forest bird occurrence as a function of the amount of early-seral broadleaf forest at landscape scales. Ecological Applications 20: 2116-2130.

5. Esseen P-A, Ehnström B, Ericson L, Sjöberg K (1997) Boreal Forests. Ecological Bulletins: 16-47.

6. McShea WJ, Rappole JH (2000) Managing the Abundance and Diversity of Breeding Bird Populations through Manipulation of Deer Populations. Conservation Biology 14: 1161-1170.

7. Côté SD, Rooney TP, Tremblay J-P, Dussault C, Waller DM (2004) Ecological impacts of deer overabundance. Annual Review of Ecology, Evolution, and Systematics 35: 113-147.

8. Ludwig GX, Alatalo RV, Helle P, Nissinen K, Siitari H (2008) Large-scale drainage and breeding success in boreal forest grouse. Journal of Applied Ecology 45: 325-333.

9. Lassau SA, Hochuli DF, Cassis G, Reid CAM (2005) Effects of habitat complexity on forest beetle diversity: do functional groups respond consistently? Diversity and Distributions 11: 73-82.

10. Sarkkola S, Hökkä H, Laiho R, Päivänen J, Penttilä T (2005) Stand structural dynamics on drained peatlands dominated by Scots pine. Forest Ecology and Management 206: 135-152.

11. Lachance D, Lavoie C, Desrochers A (2005) The impact of peatland afforestation on plant and bird diversity in southeastern Québec. Ecoscience 12: 161-171.

12. de Jong J, Humphrey JW, Smith M, Ravn HP (2008) The impact of forest management on biodiversity. EFORWOOD Project 518128. Deliverable D2.2.3 Papers on impacts of forest management on environmental services, p11-22.

13. Niemi G, Hanowski J, Helle P, Howe R, Mönkkönen M, et al. (1998) Ecological sustainability of birds in boreal forests. Conservation Ecology 2: 17.

14. Hagar JC (2007) Wildlife species associated with non-coniferous vegetation in Pacific Northwest conifer forests: A review. Forest Ecology and Management 246: 108-122.

15. Brockerhoff EG, Jactel H, Parrotta JA, Quine CP, Sayer J (2008) Plantation forests and biodiversity: oxymoron or opportunity? Biodiversity and Conservation 17: 925-951.

16. Willson MF, Comet TA (1996) Bird Communities of Northern Forests: Ecological Correlates of Diversity and Abundance in the Understory. The Condor 98: 350-362.

17. Jactel H, Nicoll BC, Branco M, Gonzalez-Olabarria JR, Grodzki W, et al. (2009) The influences of forest stand management on biotic and abiotic risks of damage. Annals of Forest Science 66.

18. Niemelä J (1999) Management in relation to disturbance in the boreal forest. Forest Ecology and Management 115: 127-134.

19. Bengtsson J, Nilsson SG, Franc A, Menozzi P (2000) Biodiversity, disturbances, ecosystem function and management of European forests. Forest Ecology and Management 132: 39-50.

20. Paillet Y, Berges L, Hjalten J, Odor P, Avon C, et al. (2010) Biodiversity Differences between Managed and Unmanaged Forests: Meta-Analysis of Species Richness in Europe. Conservation Biology 24: 101-112.

21. Wilson MW, Pithon J, Gittings T, Kelly TC, Giller PS, et al. (2006) Effects of growth stage and tree species composition on breeding bird assemblages of plantation forests: Capsule Bird species assemblages are strongly dependent on growth stage and forest structure, but do not appear to be greatly affected by tree species composition. Bird Study 53: 225-236.

22. Robles H, Ciudad C, Matthysen E (2011) Tree-cavity occurrence, cavity occupation and reproductive performance of secondary cavity-nesting birds in oak forests: The role of traditional management practices. Forest Ecology and Management 261: 1428-1435.

23. Verschuyl J, Riffell S, Miller D, Wigley TB (2011) Biodiversity response to intensive biomass production from forest thinning in North American forests - A meta-analysis. Forest Ecology and Management 261: 221-232.

24. Gil-Tena A, Brotons L, Saura S (2009) Mediterranean forest dynamics and forest bird distribution changes in the late 20th century. Global Change Biology 15: 474-485.

25. Gil-Tena A, Saura S, Brotons L (2007) Effects of forest composition and structure on bird species richness in a Mediterranean context: implications for forest ecosystem management. Forest ecology and Management 242: 470-476.
