# Supplementary material for: Quantifying the Detrimental Impacts of Land-Use and Management Change on European Forest Bird Populations
Source: PLoS One. 2013 May 21;8(5):e64552. doi: 10.1371/journal.pone.0064552 (PMC3660351; doi:10.1371/journal.pone.0064552)
Supplement: Table S4 — Quantitative scaling factors for extent of each forest change. (DOCX) [file pone.0064552.s004.docx]

**Table S4: Quantitative scaling factors for each change to forest habitat and sources of data**

| **Change to forest habitat** | **Scaling factor** | **Sources** |
| --- | --- | --- |
| Increased abundance of small predators | Change in timber yield 1980-2009 | [1,2,3] |
| Increased fire suppression | Change in area burnt 1990-2009 | [4] |
| Increased grazing pressure from domestic and wild herbivores | Change in roe deer population 1980s-2000s | [5] |
| Intensified drainage management | Change in timber yield 1980-2009 | [1,2,3] |
| Intensified soil management | Change in timber yield 1980-2009 | [1,2,3] |
| Intensified thinning | Change in timber yield 1980-2009 | [1,2,3] |
| Reduced abundance of broadleaf species | Change in timber yield 1980-2009 | [1,2,3] |
| Reduced rotation length | Change in timber yield 1980-2009 | [1,2,3] |
| Removal of deadwood | Change in timber yield 1980-2009 | [1,2,3] |
| Reduced area of broadleaf/mixed forest | Change in area of broadleaf and mixed forest  1990-2005 | [1,2] |
| Reduction in management | Change in number of people employed in forestry  1990-2010 | [2] |
| Reduced diversity of tree species | Change in timber yield 1980-2009 | [1,2,3] |
| Increased forest fires | Change in area burnt 1990-2009 | [1,2,3] |
| Loss of habitat through urbanisation | Change in urban population 1981-2010 | [6] |
| Increased selective logging | Change in timber yield 1980-2009 | [1,2,3] |

**References**

1. MCPFE (Ministerial Conference on the Protection of Forests in Europe) (2007) State of Europes’s Forests 2007. The MCPFE Report on Sustainable Forest Management in Europe. Warsaw, Poland.

2. Forest Europe, UNECE, FAO (2011) State of Europe’s Forests 2011. Status and Trends in Sustainable Forest Management in Europe.

3. UNECE (United Nations Economic Commission for Europe) Timber Database 1967 – 2007. <http://www.unece.org/forests/mis/fp-stats.html>. Accessed September 2011.

4. JRC (Joint Research Centre), European Commission, (2009) Forest Fires in Europe 2009. JRC Scientific and Technical Reports, Report No 10. Ispra, Itlay.

5. Burbaitė L, Csányi S (2009) Roe deer population and harvest changes in Europe. Estonian Journal of Ecology 58: 169-180.

6. Brinkhoff, T (2011) City Population, <http://www.citypopulation.de>. Accessed September 2011
