# Supplementary material for: Quantifying the Detrimental Impacts of Land-Use and Management Change on European Forest Bird Populations
Source: PLoS One. 2013 May 21;8(5):e64552. doi: 10.1371/journal.pone.0064552 (PMC3660351; doi:10.1371/journal.pone.0064552)
Supplement: Table S5 — Qualitative scaling factors for extent of each forest change. (DOCX) [file pone.0064552.s005.docx]

**Table S5: Survey responses from ornithological experts^1^ specialising in forest systems used for the qualitative scaling mechanism. Respondents scaled the severity of changes occurring in their country, where: 0 – not present; 1 – minor; 2 – moderate; 3 – major; and 4 – severe. (CZ – Czech Republic; EE – Estonia; FI – Finland; SE – Sweden; PT – Portugal; ES – Spain; AT – Austria; BE – Belgium; DE – Germany; CH – Switzerland; UK – United Kingdom)**

|  | East | | | | | | | | North | | South | | West | | | | | | |
| --- | --- | --- | --- | --- | --- | --- | --- | --- | --- | --- | --- | --- | --- | --- | --- | --- | --- | --- | --- |
|  | CZ | CZ | CZ | CZ | CZ | EE | EE | EE | FI | SE | PT | ES | AT | BE | DE | CH | UK | UK | UK |
| **CONIFEROUS DOMINATED FOREST** | | | |  |  |  |  |  |  |  |  |  |  |  |  |  |  |  |  |
| Increased abundance of small predators | 0 | 1 | 1 | 1 | 0 | 3 | 2 | 2 | 1 | 1 | 0 | - | 0 | - | 0 | 0 | 2 | 1 | 2 |
| Increased fire suppression | 0 | 0 | 0 | 1 | 0 | 1 | 2 | 0 | 1 | 0 | 0 | - | 0 | - | 1 | 0 | 0 | 1 | 1 |
| Increased grazing | 2 | 0 | 1 | 2 | 1 | 0 | 0 | 0 | 0 | 0 | 0 | - | 0 | - | 2 | 0 | 1 | 3 | 2 |
| Intensified drainage management | 2 | 3 | 2 | 1 | 2 | 3 | 4 | 1 | 2 | 0 | 0 | - | 0 | - | 1 | 0 | 1 | 2 | 2 |
| Intensified soil management | 1 | 2 | 1 | 0 | 2 | 2 | 1 | 0 | 1 | 1 | 0 | - | 1 | - | 0 | 0 | 1 | 2 | 2 |
| Intensive thinning | 1 | 0 | 1 | 1 | 3 | 2 | 4 | 0 | 1 | 1 | 0 | - | 2 | - | 3 | 1 | 0 | 0 | 0 |
| Reduced broadleaf component | 2 | 3 | 1 | 1 | 4 | 2 | 1 | 1 | 2 | 2 | 3 | - | 2 | - | 3 | 1 | 0 | 3 | 2 |
| Reduced rotation length | 0 | 1 | 3 | 2 | 4 | 3 | 4 | 2 | 4 | 2 | 0 | - | 3 | - | 3 | 2 | 1 | 2 | 2 |
| Removal of dead wood | 1 | 1 | 3 | 2 | 4 | 4 | 3 | 3 | 4 | 2 | 1 | - | 2 | - | 4 | 2 | 0 | 1 | 1 |
| **BROADLEAF AND MIXED FOREST** | | | |  |  |  |  |  |  |  |  |  |  |  |  |  |  |  |  |
| Increased abundance of small predators | 0 | 2 | 1 | 1 | 0 | 3 | 2 | 2 | 1 | 1 | 0 | - | 0 | 1 | 0 | 0 | 2 | 1 | 2 |
| Increased grazing | 2 | 0 | 1 | 2 | 2 | 0 | 0 | 0 | 0 | 1 | 1 | - | 0 | 3 | 1 | 1 | 3 | 4 | 4 |
| Intensified drainage management | 1 | 3 | 2 | 1 | 2 | 3 | 4 | 0 | 1 | 0 | 0 | - | 1 | 1 | 0 | 0 | 1 | 2 | 2 |
| Reduced broadleaf/mixed area | 3 | 1 | 1 | 2 | 4 | 2 | 2 | 2 | 2 | 1 | 1 | - | 2 | 2 | 2 | 2 | 0 | 1 | 1 |
| Reduced grazing | 0 | 0 | 0 | 1 | 0 | 0 | 0 | 0 | 2 | 0 | 1 | - | 0 | 0 | 1 | 0 | 2 | 2 | 2 |
| Reduced management | 0 | 1 | 1 | 0 | 0 | 0 | 0 | 1 | 1 | 0 | 2 | - | 1 | 1 | 1 | 2 | 3 | 4 | 4 |
| Reduced rotation length | 0 | 3 | 3 | 2 | 4 | 3 | 4 | 2 | 4 | 2 | 0 | - | 3 | 1 | 3 | 2 | 1 | 2 | 2 |
| Reduced tree species diversity | 3 | 2 | 2 | 2 | 4 | 2 | 2 | 3 | 2 | 2 | 1 | - | 3 | 0 | 4 | 0 | 0 | 4 | 2 |
| Removal of dead wood | 1 | 1 | 3 | 2 | 4 | 4 | 3 | 3 | 4 | 2 | 0 | - | 3 | 2 | 4 | 1 | 0 | 1 | 1 |
| **MEDITERRANEAN FOREST** | | |  |  |  |  |  |  |  |  |  |  |  |  |  |  |  |  |  |
| Increased forest fires | - | - | - | - | - | - | - | - | - | - | 3 | 3 | - | - | - | - | - | - | - |
| Increased grazing | - | - | - | - | - | - | - | - | - | - | 2 | 1 | - | - | - | - | - | - | - |
| Loss of habitat through urbanisation | - | - | - | - | - | - | - | - | - | - | 1 | 2 | - | - | - | - | - | - | - |
| Reduced management | - | - | - | - | - | - | - | - | - | - | 1 | 3 | - | - | - | - | - | - | - |
| Selective logging | - | - | - | - | - | - | - | - | - | - | 1 | 2 | - | - | - | - | - | - | - |

^1^Ornithological experts specialising in forest habitats were a subset of those acknowledged in Table S1
