# Supplementary material for: Quantifying the Detrimental Impacts of Land-Use and Management Change on European Forest Bird Populations
Source: PLoS One. 2013 May 21;8(5):e64552. doi: 10.1371/journal.pone.0064552 (PMC3660351; doi:10.1371/journal.pone.0064552)
Supplement: Appendix S1 — Overview of risk assessment process. (DOCX) [file pone.0064552.s006.docx]

**Appendix S1:**

**Overview of risk assessment process – see [1] for further details**

We defined the potential risk of forest change *x* to species *y* as the degree of coincidence between the environmental impacts of that change and the ecological requirements of that species, adjusted for the species’ ecological resilience. Ecological resilience is quantified by the breadth of its ecological requirements and its reliance on forest habitat. Our risk assessment framework assumes that the major sources of risk to forest birds will be reduced food abundance and reduced nesting success. Forest change *x* will impact food abundance if it causes a change in foraging habitat availability and/or a change in prey abundance in the existing foraging habitat. It will impact nesting success if it causes a change in nesting habitat availability and/or a reduction in nest success in the existing nesting habitat. Thus:

Risk score = (*D*_t_ + *N*_t_) / *R* (eqn S.1)

where *D_t_* represents the proportion of foraging habitats and prey items used by species *y* that are affected by forest change *x*, *N_t_* represents the proportion of nesting habitats used by species *y* that are affected by forest change *x* and *R* = reliance of species *y* on forest habitat. Species deemed to have a major reliance on forest habitats score 1, those with a moderate reliance score 2 and those with a minor reliance score 3.

Model structure

The above risk scoring system assumes an equal weighting for all sources of risk and that effects are additive across risk sources. The derived risk score for a species is the sum of its diet-related risk and nest-related risk across all forest changes, divided by its reliance score. The separate components of the risk score for a species are calculated using the following equations:

*D_t_* = *A_s_*/(*D_s_***F_s_*) + *A_w_*/(*D_w_***F_w_*) + *B_s_*/*F_s_* + *B_w_*/*F_w_* (eqn S.2)

Loss of prey in existing habitat

Loss of foraging habitat

where *D_t_* is as defined in equation (eqn S.1), *A_s_* = number of points of coincidence between potential impact on and the species’ use of diet components in summer, *A_w_* = number of points of coincidence between potential impact on and the species’ use of diet components in winter, *B_s_* = number of points of coincidence between potential impact on and the species’ use of foraging habitat components in summer, *B_w_* = number of points of coincidence between potential impact on and the species’ use of foraging habitat components in winter, *D_s_* = number of diet components used by the species in summer, *D_w_* = number of diet components used by the species in winter, *F_s_* = number of foraging habitat components used by the species in summer, *F_w_* = number of foraging habitat components used by the species in winter. The products of *D_s_* and *F_s_* and of *D_w_* and *F_w_* are used because the model assumes each dietary item used is available in each foraging habitat used.

*N_t_* = *C_1_*/*N* + *C_2_*/*N* (eqn S.3)

Reduced nest success in existing habitat

Loss of nesting habitat

where *N_t_* is as defined in equation (eqn S.1), *C_1_* and *C_2_* = number of points of coincidence between potential impact on and the species’ use of nesting habitat components if impact is through reduced success in existing habitat and loss of habitat respectively and *N* = number of nesting habitat components used by the species.

The maximum possible risk score generated by a single forest change is six. This score would be calculated if a species has a major reliance on forest and if the forest change causes a reduction in the availability of all foraging habitats used (summer and winter), a reduction of all diet components used (summer and winter) in the remaining foraging habitat, a reduction in the availability of all nesting habitats used and a reduction in nest success in the remaining nesting habitat.

**Reference**

1. Butler SJ, Vickery JA, Norris K (2007) Farmland biodiversity and the footprint of agriculture. Science 315: 381-384.
